# Study of Interactions of an Anticancer Drug Neratinib With Bovine Serum Albumin: Spectroscopic and Molecular Docking Approach

**DOI:** 10.3389/fchem.2018.00047

**Published:** 2018-03-07

**Authors:** Tanveer A. Wani, Ahmed H. Bakheit, M. A. Abounassif, Seema Zargar

**Affiliations:** ^1^Department of Pharmaceutical Chemistry, College of Pharmacy, King Saud University, Riyadh, Saudi Arabia; ^2^Department of Chemistry, Faculty of Science and Technology, Al-Neelain University, Khartoum, Sudan; ^3^Department of Biochemistry, College of Science, King Saud University, Riyadh, Saudi Arabia

**Keywords:** bovine serum albumin, neratinib, human serum albumin, fluorescence, quenching

## Abstract

Binding of therapeutic agents to plasma proteins, particularly to serum albumin, provides valuable information in the drug development. This study was designed to evaluate the binding interaction of neratinib with bovine serum albumin (BSA). Neratinib blocks HER2 signaling and is effective in trastuzumab-resistant breast cancer treatment. Spectrofluorometric, UV spectrophotometric, and fourier transform infrared (FT-IR) and molecular docking experiments were performed to study this interaction. The fluorescence of BSA is attributed to the presence of tryptophan (Trp) residues. The fluorescence of BSA in presence of neratinib was studied using the excitation wavelength of 280 nm and the emission was measured at 300-500 nm at three different temperatures. Neratinib quenched the BSA intrinsic fluorescence by static mechanism. A complex formation occurred due to the interaction leading to BSA absorption shift. The fluorescence, UV- absorption, three dimensional fluorescence and FT-IR data showed conformational changes occurred in BSA after interaction with neratinib. The binding constant values decreased as the temperature increased suggesting an instable complex formation at high temperature. Site I (sub-domain IIA) was observed as the principal binding site for neratinib. Hydrogen bonding and Van der Waals forces were suggested to be involved in the BSA-neratinib interaction due to the negative values of entropy and enthalpy changes.

## Introduction

**Figure 1:**
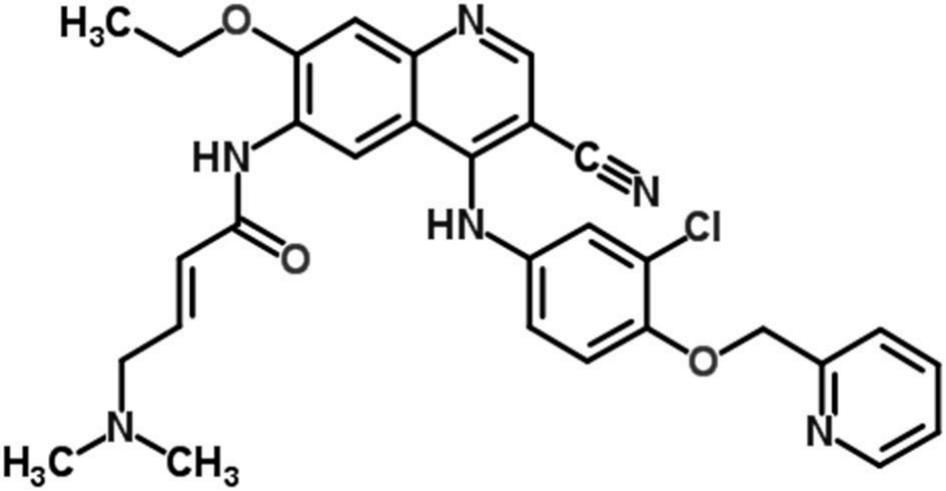
Chemical structure of neratinib.

Neratinib, a tyrosine kinase inhibitor, is used in trastuzumab-resistant breast cancer treatment as an alternative to block HER2 signaling (Figure 1; Burstein et al., 2010; Iqbal and Iqbal, 2014; Wani et al., 2015). Neratinib has been recently approved by United States FDA for use in early stage HER2-overexpressed/amplified breast cancer (Bose and Ozer, 2009; Feldinger and Kong, 2015; Kourie et al., 2016; US Food and Drug Administration, 2018).

Plasma proteins act as carriers for transportation of drugs and other compounds. Amongst the various plasma proteins, serum albumin is the most abundant protein and it plays a vital role in transportation of drug ligands (Jahanban-Esfahlan et al., 2015; Wani et al., 2017b,c). Several tyrosine kinase inhibitors have been studied for their interaction with bovine serum protein (BSA) (Shen et al., 2015) and in this study, the interaction of neratinib with BSA was explored. BSA was selected for studying the interaction owing to its structural similarity to human serum albumin (HSA), low procurement cost and ready availability (He and Carter, 1992; Chi et al., 2010). So far, studies on the interaction between plasma proteins and neratinib only focused on the characterization of neratinib covalent binding with serum albumin and reversible covalent binding of neratinib with plasma proteins (Chandrasekaran et al., 2010; Wang et al., 2010). The BSA contains 583 amino acids and three homologous domains. These homologous I, II, and III domains are connected by disulfide bonds. Two tryptophan residues namely Trp-134 and Trp-212, are present in BSA molecule and have intrinsic fluorescence (Kragh-Hansen, 1981). The pharmacokinetics parameters of distribution, transportation and excretion of small ligands depend on the noncovalent binding interactions of drug ligands to proteins. Exploration of the interaction mechanism between the drug ligands with BSA is of great interest (Berezhkovskiy, 2007; Chamani and Heshmati, 2008; Xiao et al., 2011; Khorsand Ahmadi et al., 2015; Marouzi et al., 2017).

The interaction between neratinib and serum albumin was explored in this study. Multispectroscopic (UV-vis absorption, fluorescence, FT-IR) along with computational approaches were used to study the binding interaction. The parameters under study included binding site involvement, complex formation and binding energies of neratinib with BSA. The molecular docking data were corroborated with experimental results to obtain a better understanding of the mechanisms involved in the interaction.

## Methods

### Chemicals and reagents

Bovine serum albumin (BSA) was procured from Sisco Research Laboratories, India. Neratinib was obtained from Selleckchem, USA. Phenylbutazone and ibuprofen were purchased through National Scientific Company, Saudi Arabia. The stock solutions for neratinib, BSA, phenylbutazone and ibuprofen were prepared as per their molecular weight. Phosphate buffer pH 7.4 was used for preparation of BSA stock solution of 1.5 × 10^−6^ M. Neratinib was dissolved in 500 μL dimethyl sulphoxide and then diluted with phosphate buffer pH 7.4 to get a stock concentration of 1.8 × 10^−3^ M. The stock concentration was further diluted with the buffer to obtain working standard solutions in the range of 3.8 × 10^−5^ and 5.2 × 10^−4^ M. The stock solutions of ibuprofen and phenylbutazone were prepared in methanol and then diluted with the phosphate buffer. The deionized water was obtained from a Flex Type-IV instrument from Elga Lab Water, UK.

### Fluorescence spectra measurement

The fluorescence analysis was carried out using a JASCO FP-8200 spectrofluorometer (Japan). The chosen excitation wavelength was 280 nm and the emission fluorescence was attained within the 300–500 nm range. BSA solution 1.5 × 10^−6^ M was titrated with different neratinib concentrations (0, 1.5 × 10^−6^, …., 2.11 × 10^−5^ M) and the fluorescence measurements were carried out at the temperatures of 298, 303, and 308 K. These two solutions were mixed in a ratio of 1:1 *v/v*. Thus, the concentrations measured were half of the initial concentrations of either BSA or neratinib. The fluorescence intensity (FI) might decrease due to inner filter effect since a compound present in the solution might absorb in the ultraviolet region near the excitation/emission wavelength. Therefore, the correction of FI was done for studying the neratinib–BSA interaction using the following equation:

$$\begin{array}{l} Fcor=Fobs\times e^{\left(Aex+Aem\right)/2} \end{array}$$

Where, *Fcor* and *Fobs* denote corrected fluorescence intensity and measured fluorescence intensity respectively; and *Aex* and *Aem* are the modified absorbance values of the protein upon ligand addition at the excitation and emission wavelengths, respectively.

### Synchronous fluorescence spectra measurement

The synchronous fluorescence spectra were studied for conformational changes that could occur in BSA at 298 K (room temperature). Scanning intervals Δλ (Δλ = λem-λex) of 15 and 60 nm characterize the tyrosine and tryptophan residues, respectively.

### FT-IR spectra measurement

A Bruker Alpha II FT-IR spectrometer (USA) coupled with the OPUS software was used. The spectra (spectral resolution 2 cm^−1^; 24 scans) obtained were converted into absorbance. The spectra for the buffer and BSA solution in buffer were obtained, and the spectrum of buffer solution was subtracted from the BSA solution to get FT-IR spectra of BSA. Similarly, the BSA-neratinib solution was prepared and the spectra for the free neratinib was subtracted from the bound form. The FT-IR results provided evidence of possible conformational changes in the protein molecule.

### Site probe experiment

Site probe experiments were also conducted to determine the binding site involved in the interaction. Different concentrations of neratinib were added to equimolar concentrations of site probes (phenylbutazone or ibuprofen) and BSA; the FI was then determined at room temperature (298 K) and excitation wavelength of 280 nm.

### UV–visible spectra measurement

The UV-Visible absorption spectra were attained in the range of 200–400 nm for BSA, neratinib and BSA-neratinib complex at room temperature (298 K) with a UV-1800 spectrophotometer (Shimadzu, Japan). The BSA-neratinib spectra were acquired by keeping BSA concentration constant (1.5 μM) and varying neratinib concentration.

### Molecular docking

Molecular docking analysis was performed to studythe interaction between neratinib and BSA. The docking was performed on Molecular Operating Environment (MOE-2014). The structure for neratinib was drawn in the MOE, whereas the BSA crystalline protein structure was obtained from protein data bank (pdb) with the pdb code number 4OR0 (http://www.rcsb.org). Chain A of the BSA molecule was selected for the docking analysis due to the fact that BSA exist as a homodimer of two chains. Both protein receptors and ligands were protonated when prepared; and the energy minimization was performed with the default parameters of Force field MMFF94X, eps = r and cut off (8–10). The docking parameters used in the analysis were kept as default with Triangle Matcher. The rescoring function 1 was set as London dG and the rescoring function 2 was set as GBVI/WSA dG along with 10 conformation generations in order to fit the binding groove. mdb output file was generated for further analysis and evaluation of neratinib–BSA interaction. The active binding site that might be involved in the interaction was obtained from the site specific probe experiments (Jahanban-Esfahlan et al., 2015; Wani et al., 2017b,c). RMSD (root mean square deviation) parameters were used to select the most suitable interaction of BSA with neratinib.

## Results

### Fluorescence quenching

**Figure 2:**
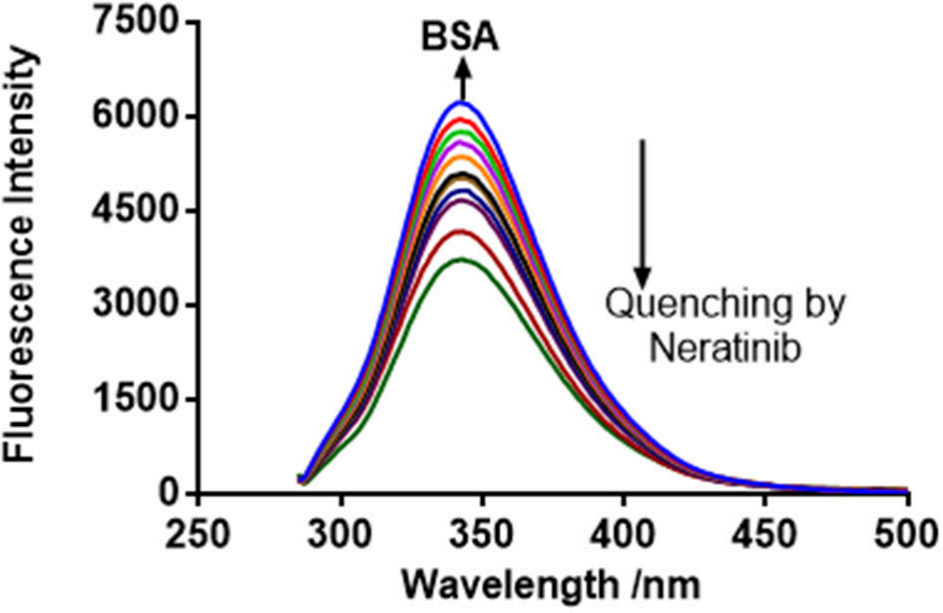
BSA fluorescence quenching spectra in presence of neratinib at 298 K, λex = 280 nm.

The FI of BSA and BSA-neratinib complex were recorded with excitation at 280 nm and emission in the range of 300–500 nm. The BSA concentration was kept constant whereas, the concentration of neratinib was varied. A decrease in FI was observed with increasing neratinib concentration. This was attributed to the quenching of fluorescence by BSA because of the formation of a non-fluorescent complex between neratinib and BSA (Figure 2). The quenching data was analyzed using the Stern-Volmer equation:

$$\begin{array}{l} \frac{F}{F_{0}}=1+K_{sv}\left[Q\right]=1+K_{q}\;\tau_{0}\;\left[Q\right] \end{array}$$

**Figure 3:**
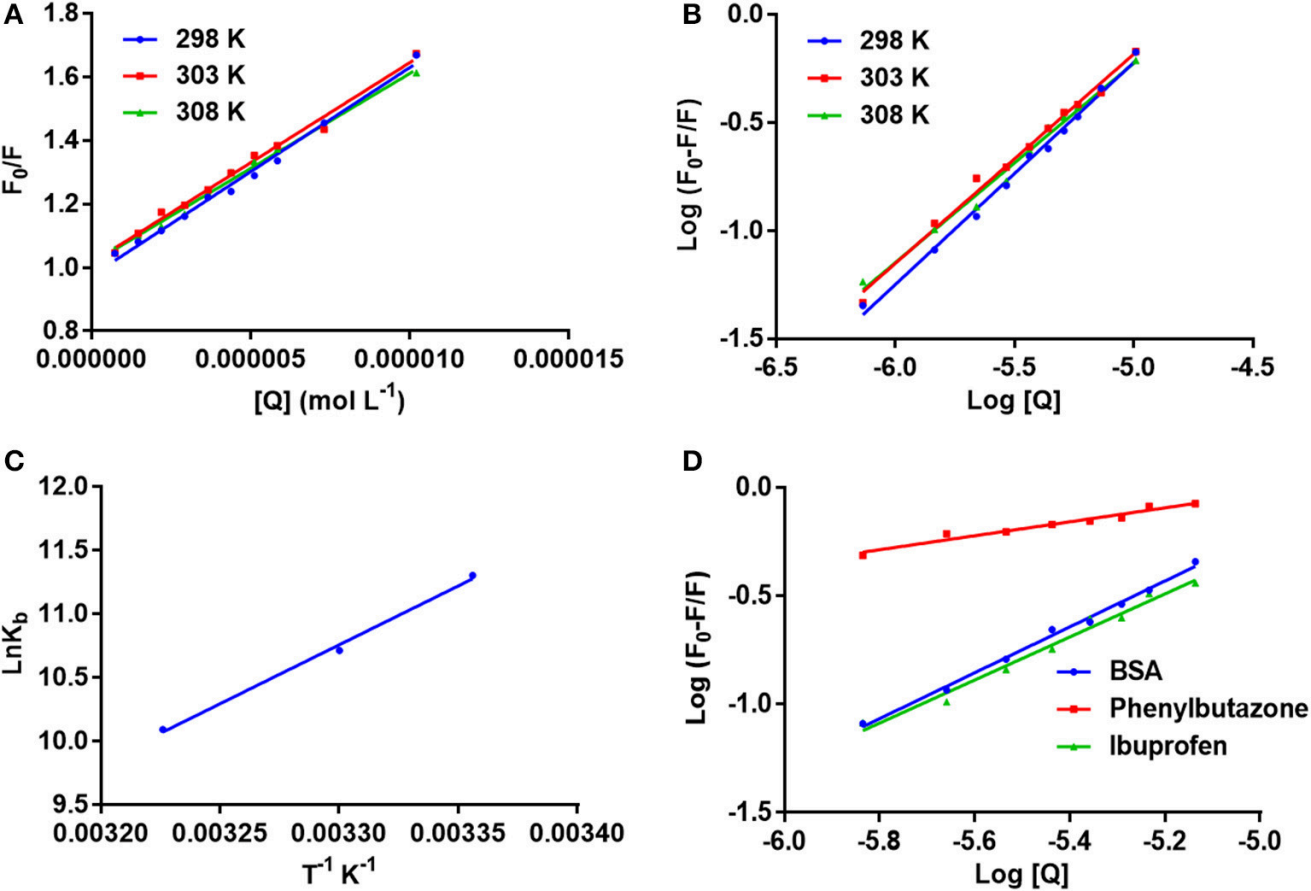
**(A)** The stern–Volmer curves for the quenching of BSA by neratinib at 298/303/308 K; **(B)** The plot of log[(F_0_-F)/F] vs. log[Q] for quenching process of neratinib with BSA at 298/303/308 K; **(C)** Van't Hoff plots for the binding interaction of neratinib with BSA; **(D)** The plot of log[(F_0_-F)/F] vs. log[Q] for quenching process of neratinib with BSA in presence of site markers phenylbutazone and ibuprofen at 298 K.

**Figure 4:**
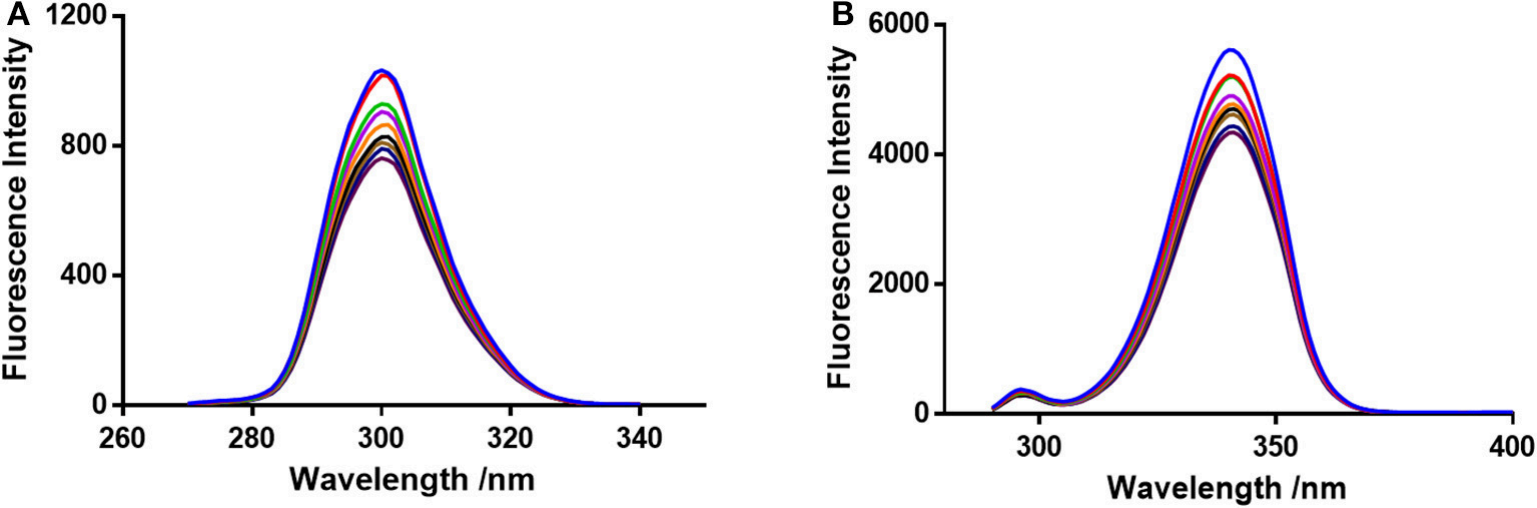
Synchronous fluorescence spectroscopy of BSA and neratinib at 298 K **(A)** Δλ = 15 nm and **(B)** Δλ = 60 nm.

**Figure 5:**
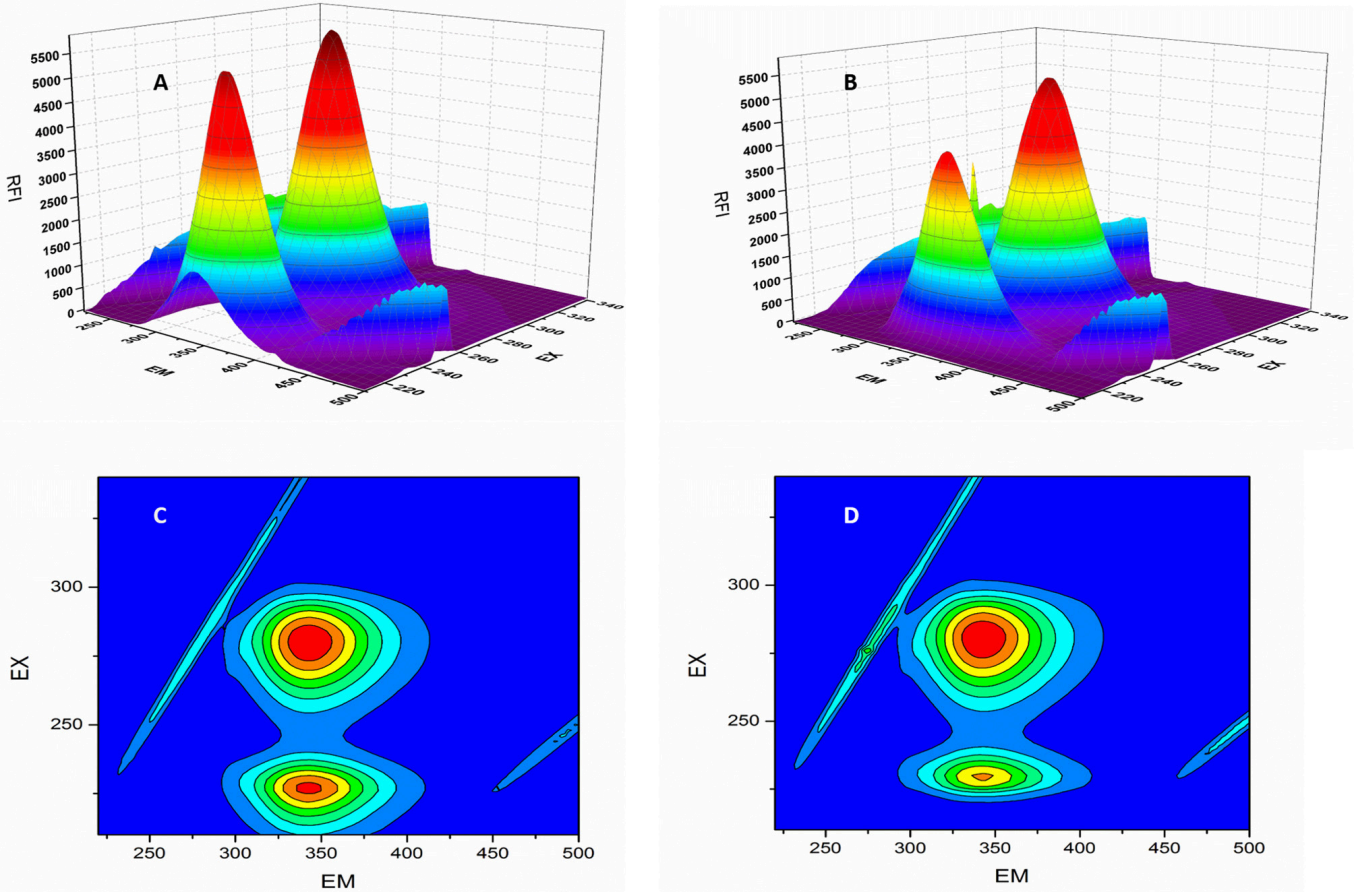
3D-spectroflurometric analysis of BSA and neratinib–BSA system. **(A,B)** are normal 3D spectra and **(C,D)** represent the contour plot of FI.

*F*_0_ and *F* represent the FIs in absence and presence of neratinib; K*sv:* Stern-Volmer quenching constant; [*Q*]: quencher concentration; K_q:_ quenching rate constant; τ_0:_ fluorophore's lifetime devoid of quencher and is valued 10^−8^ for a biopolymer (Lakowicz and Weber, 1973). The values obtained for Ksv at the three different temperatures are presented in Table 1 (Figure 3). During the synchronous fluorescence experiments, a stronger quenching of FI was observed for tryptophan residues Δλ = 60 nm compared to tyrosine residues Δλ = 15 nm indicating the contribution of tryptophan in the intrinsic fluorescence of BSA (Figure 4). Also a red shift equal to 1 nm was observed for tryptophan residue. The 3D (3-dimensional) spectrofluorometric analysis of BSA and BSA-neratinib complex (Figure 5) was performed indicating changes in the BSA conformation after addition of neratinib.

**Table 1:** Stern–Volmer quenching constants (K_SV_) and bimolecular quenching rate constant (Kq) for the binding of neratinib to BSA at three different temperatures.

**T(K)**	**R**	**Ksv ± SD × 10^4^ (L mol^−1^)**	**Kq × 10^12^ (L mol^−1^s^−1^)**
298	0.9918	6.54 ± 0.31	6.54
303	0.9907	6.28 ± 0.18	6.28
308	0.9935	5.96 ± 0.37	5.96

### Binding constant

Small drug ligands interact with proteins binding sites independently and the equilibrium among the free and bound molecules is represented by the following equation (He et al., 2010):

$$\begin{array}{l} \log\frac{\left(F_{0}-F\right)}{F}=\text{nlog }K_{b}\pm n\text{ log}\left[\frac{1}{\left[Q\right]-\frac{\left(F_{0}-F\right)\left[P\right]}{F_{0}}}\right] \end{array}$$

Where K_*b*_ is binding constant and n is binding site number; [*Q*] and [*P*] are the total concentrations of quencher and protein. A plot between log (*F*_0_-*F*)/*F* vs. log {1/([*Q*]–(*F*_0_-*F*) [*P*]/*F*_0_)} is used to calculate the binding constant (intercept) and number of binding sites (slope). The binding constants and number of binding sites were determined at all the three temperatures and are presented in Table 2. The number of binding sites were found equal to unity. The binding constant obtained for BSA-neratinib complex was found to be 8.1 × 10^4^, whereas, in presence of phenylbutazone and ibuprofen were found to be 0.38 × 10^2^ and 4.8 × 10^4^, respectively (Figure 3).

**Table 2:** Binding and thermodynamic parameters of binding between neratinib and BSA.

**T(K)**	**R**	**Log K_b_ ± SD**	**K_b_ ± SD × 10^4^ (L mol^−1^)**	**n**	**ΔG (kJ mol^−1^)**	**ΔH (kJ mol^−1^)**	**ΔS (Jmol^−1^·K^−1^)**
298	0.9938	4.909	8.10 ± 0.20	1.02	−27.93	−76.9	−164
303	0.9905	4.653	4.50 ± 0.24	0.96	−27.11		
308	0.9902	4.383	2.42 ± 0.11	0.92	−25.96		

### Binding mode

The binding mode is established based on the thermodynamic parameters that include enthalpy change (ΔH^0^), entropy change (ΔS^0^) and free energy change (ΔG^0^). The thermodynamic parameters are given in Table 2. Figure 3 represents the van't Hoff plot for neratinib and BSA interaction.

## Discussion

### Neratinib binding to the serum albumins

Fluorescence spectroscopy acts as a tool for investigation of the interaction between biological macromolecules (proteins) and small drug ligands. The interaction can be studied in terms of the mechanism involved in binding interaction, binding constants, etc. The FI can get reduced due to several molecular interactions that may include excited-state reactions, complex formations, energy transfer and molecular rearrangements. This decrease in the FI is known as fluorescence quenching. The type of quenching involved (static or dynamic) is derived from the linearity of the Stern-Volmer plot between F_0_/F vs. [Q] (Figure 3). The Stern-Volmer plot alone cannot give sufficient information about the nature of quenching involved in the interaction. Thus, other evidences are still required for its determination. The change in temperature is used as a tool to investigate and distinguish between the static and dynamic quenching that may be involved in ligand-BSA interaction. The Ksv value decreases at higher temperature in static quenching, and vice versa in case of dynamic quenching. These results infer that a static quenching and complex formation could occur between neratinib and BSA. It was further supported by the quenching rate constants obtained (Table 1). The quenching constant for collision quenching can achieve a maximum value 2 × 10^10^ M^−1^ S^−1^ for biopolymers. Our quenching constant values were much higher than those obtained by scattered procedure clearly showing the involvement of static quenching in the BSA-neratinib interaction (Shi et al., 2014; Wani et al., 2017a).

The synchronous fluorescence spectrophotometric experiments were performed to obtain information regarding the microenvironment present in the immediate neighborhood of chromosphere molecules. The conformational changes were reflected by changes in the maximum emission wavelength. A higher quenching and red shift of 1 nm was observed for tryptophan residue suggesting an increase in polarity of the surrounding environment (Figure 4). Therefore, it was concluded that the BSA conformation changes upon interaction of neratinib with BSA (Albert et al., 2006; Meti et al., 2015).

In the 3-dimensional spectral analysis for BSA in presence of neratinib, two peaks were found namely Peak 1 and Peak 2 (Figure 5). Peak 1 was found at the excitation wavelength of 230 nm and emission wavelength of 344 nm. Peak 1 is formed due to π-π^*^transition of polypeptide structures present in the BSA molecule. Peak 2 was found at the excitation and emission wavelength of 280 and 342 nm, respectively. Tryptophan and tyrosine residues are responsible for the formation of Peak 2. A sharp decrease in the FI of BSA was witnessed after addition of neratinib meaning that fluorescence quenching occured. A sparse spectrum in the contour plot (Figures 5C,D) was observed for BSA in presence of neratinib, which confirms the occurrence of conformational changes in BSA after neratinib addition.

A decrease in the binding constants was noticed as the temperature increased indicating the instability of BSA-neratinib complex. Furthermore, the number of binding sites was found to be equal to 1, indicating a single class of binding sites on BSA.

Site specific probes, phenylbutazone and ibuprofen, were used for determination of the binding sites present on BSA (Hu et al., 2004). A decrease in the values of binding constants was observed in presence of drug site probes. Phenylbutazone caused a greater reduction in the binding constant compared to ibuprofen inferring Site I as the binding site for neratinib (Figure 3).

### Types of interaction force between BSA with neratinib

The complex formation relies on the thermodynamic process due to the fact that binding constants are temperature-dependent. The thermodynamic processes help characterize the kind of forces engaged among BSA and neratinib (Ni et al., 2008). The forces that might be involved in binding small ligands to proteins include hydrogen bonds and Van der Waals forces, hydrophobic interaction or electrostatic forces. The binding mode is established based on the thermodynamic parameters that include enthalpy change (ΔH^0^), entropy change (ΔS^0^), and free energy change (ΔG^0^). The thermodynamic parameters were evaluated by the following equations:

$$\begin{array}{r} \text{lnK}b=-\frac{\Delta H^{0}}{RT}+\frac{\Delta S^{0}}{R}\\ \Delta G^{0}=\Delta H^{0}-T\Delta S^{0}=-RT\text{ln K}b \end{array}$$

K_*b*_ and *R* represent the binding constant and universal gas constant, respectively. The negative (–) Δ*H*^0^ and Δ*S*^0^ indicate the presence of hydrogen bonding and Van der Waals forces between BSA and neratinib. Moreover, (−Δ*H*^0^) cannot occur during electrostatic interactions since these interactions occur when Δ*H*^0^ is either very small or almost zero (Ross and Subramanian, 1981; Ni et al., 2008). Figure 3, represents the van't Hoff plot for neratinib and BSA interaction. The spontaneous interaction between BSA and neratinib is indicated by (−Δ*G*^0^) value. Both the enthalpy change and entropy change acquired negative values in the neratinib-BSA interaction, suggesting an enthalpy-driven interaction and the entropy value reported as negative number indicates its unfavorability for the binding process.

### UV–vis absorption studies

**Figure 6:**
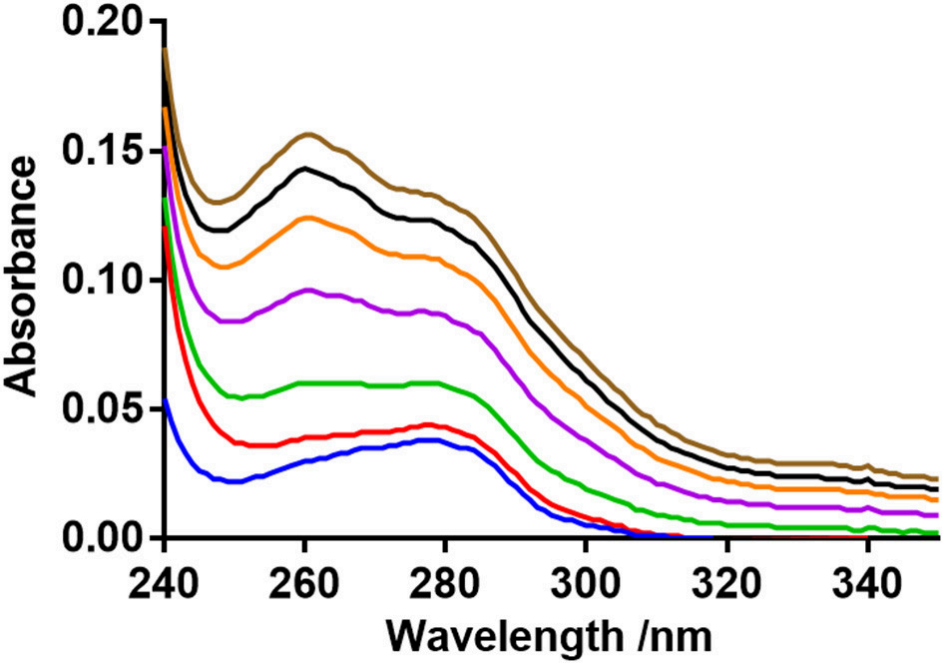
BSA UV-absorption spectra in presence of neratinib.

The UV–vis absorption spectra suggests a complex formation occurred between BSA and neratinib (Figure 6). An increase in the absorption intensity of BSA was observed with higher neratinib concentrations. The complex formation between BSA and neratinib is further confirmed as a blue shift was observed in the λmax of BSA (Kandagal et al., 2006; Peng et al., 2015).

### FT-IR studies

**Figure 7:**
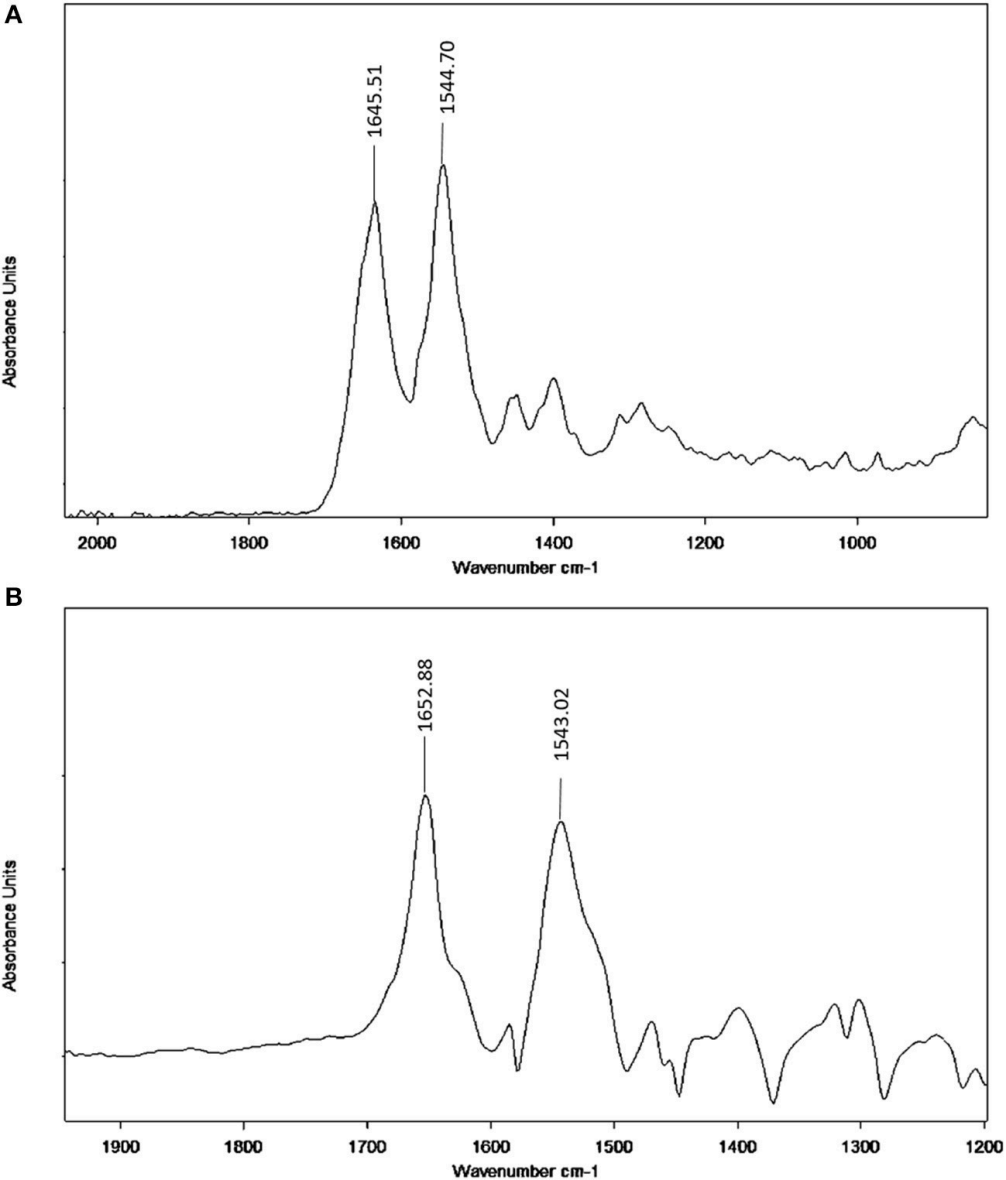
FT-IR spectra **(A)** Free BSA in aqueous solution; **(B)** Difference spectra obtained by subtracting the spectrum of the neratinib-free form from that of the neratinib-bound form.

Infrared spectroscopy is used to investigate the secondary structures and dynamics of protein. The band frequencies as a result of amide I, II, and III vibrations in the IR region provide information about the secondary protein structure (i.e., the amide I band 1,600–1,700 cm^−1^ and amide II band 1,548 cm^−1^). The information provided by amide I is more valuable due to its sensitivity to protein structure change than amide II. Figure 7 provides information regarding the changes in BSA after neratinib addition. It is clear that there were a shift of peak occurred in amide I from 1645.51 to 1652.88 cm^−1^ and a slight shift in amide II peak from 1544.70 to 1543.02 cm^−1^, suggesting a change in the secondary structure of BSA after interaction with neratinib.

### Molecular simulation studies

**Figure 8:**
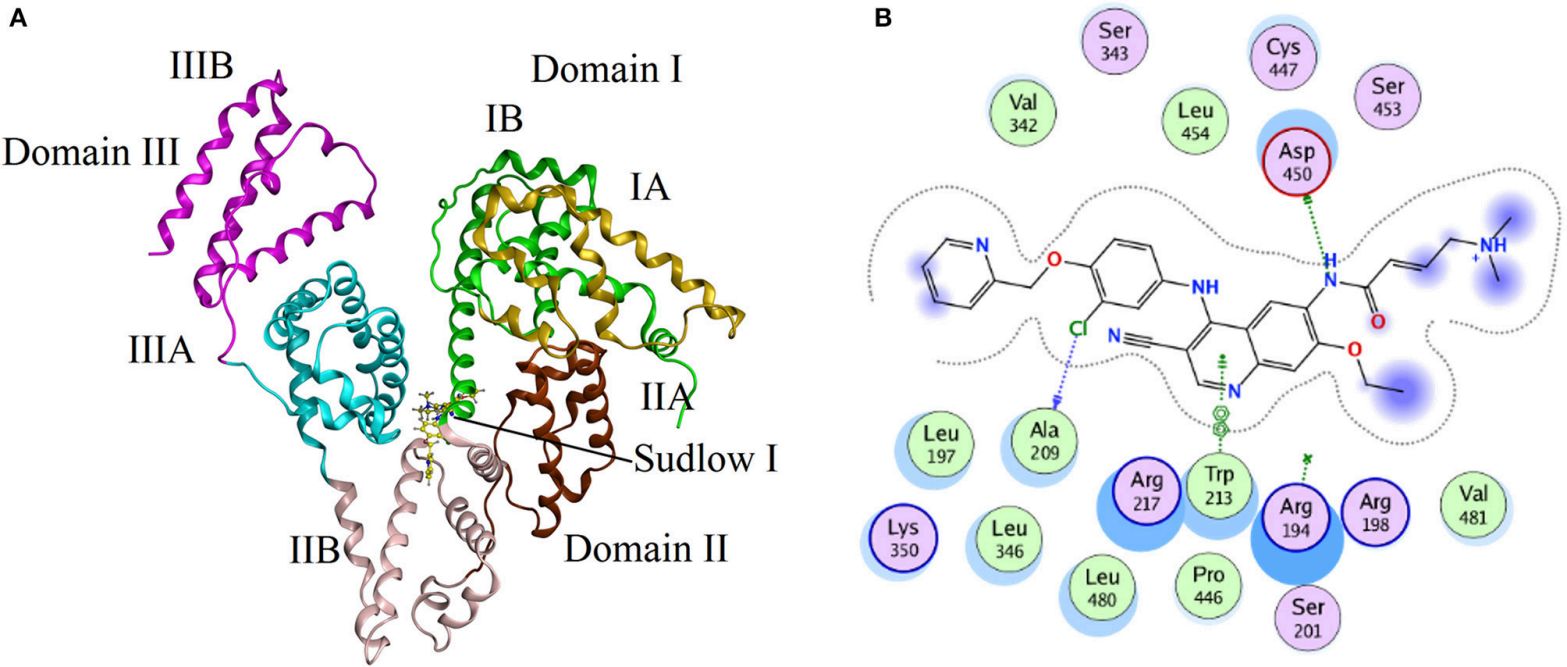
**(A)** The docking conformation of neratinib-BSA complex with lowest energy; **(B)** Represents the amino acid residues that surround neratinib.

Molecular docking experiments were performed to understand the interaction between neratinib and BSA. The docking experiments further supported spectrophotometric and spectrofluorometric data (Ali et al., 2010; Shahabadi and Fili, 2014). In molecular docking studies, the ligand gets tied to the binding pocket of the protein in different positions thus providing valuable information on the binding site and mode. The two binding sites present on BSA protein are designated as Site I and Site II, and are present in sub-domains IIA and IIIA, respectively. The site probe experiment revealed site I as the binding site for neratinib which was further confirmed by the docking results. The sub-domains IIA of site 1 was analyzed with varied conformational adaptations and the least possible BSA-neratinib complex energies were obtained. Figure 8A represents the finest conformation of neratinib-BSA complex. It is evident that neratinib interacted with Trp-213 through pi-pi interaction and with Asp-450 and Ala-209 by hydrogen bonds (Figure 8B). It was reported that neratinib forms a reversible covalent bond with Lys-190 of HSA. Neratinib contains a 4-(dimethylamino) crotonamide Michael acceptor and a covalent bond is formed between ε-amine of lysine of HSA and β-carbon of the amide functional group of neratinib. The covalent bond formed between neratinib and HSA is dependent on temperature, pH and time, and is independent of neratinib concentration (Chandrasekaran et al., 2010; Wang et al., 2010). The peptide LDELRDEGKASSAK is unique to human and monkey albumin; and neratinib binds to this peptide covalently. It has also been reported that neratinib does not bind covalently to plasma proteins from other species like dogs, rabbits and rodents as the sequence of amino acid residues from 182 to 195 in the albumin of these species is different than that in monkey and humans. The amino acid sequence of residues in BSA from 182 to 195 is ETMREKVLTSSARQ, meaning that BSA cannot bind covalently to neratinib due to this variation (Wang et al., 2010). The binding energy of neratinib-BSA complex at Site I by molecular docking was found to be −24.12 kj mol^−1^, which is in an agreement with the binding energy of −27.93 kj mol^−1^ found experimentally at 298 K. On the basis of experimental and docking results, it is concluded that hydrophobic (pi-pi interaction) and hydrophilic (hydrogen bonding) were involved in the BSA-neratinib complex stabilization.

## Conclusion

Neratinib approved for use in early stage HER2-overexpressed/amplified breast cancer was investigated for its interaction with BSA. The site probe and molecular docking experimental results established that neratinib binds to the site I, subdomain IIA of BSA. The fluorescence quenching, synchronous fluorescence, UV and FT-IR data together with the docking studies confirmed the formation of a complex between BSA and neratinib. Van der Waals forces and hydrogen bonding were found to be involved in the BSA-neratinib interaction in a enthalpy-driven manner. Based on our findings, the pharmacological and biochemical aspects involved in the BSA-neratinib interaction could be better understood.

## Author contributions

Conceived and designed the experiments: TW and MA. Performed the experiments: AB and SZ. Analyzed the data: AB and TW. Contributed reagents, materials, analysis tools: SZ, MA, and TW. Wrote the paper: TW and SZ.

### Conflict of interest statement

The authors declare that the research was conducted in the absence of any commercial or financial relationships that could be construed as a potential conflict of interest.
